# New Literature

**DOI:** 10.1155/S1463924678000152

**Published:** 1978


### New Literature

### FORTRAN IV subroutine

The US National Bureau of Standards has published *Computer Science and Technology: FORTRAN IV Enhanced Character Graphics* by N. M. Wolcott. This describes a FORTRAN IV subroutine which allows the drawing of six styles of alphabetic characters, three styles of numbers, and 48 special mathematical symbols from the enhanced graphic character set of Hershey. Twenty-two symbols for graph plotting are also provided. Output is by linkage to an external subroutine PLOT. The program requires a computer which can accommodate a 30 bit word length. This publication is available at $2.30 from the Superintendent of Documents, US Government Printing Office, Washington, DC 20402, USA. Quote SD stock number 003-003-01921-6.

### Controlling your robot

Industrial robot designers and computer hobbyists interested in robot systems will find a wealth of useful information on computer controls for their robots in a new publication released by the US National Bureau of Standards (NBS). *An Architecture for a Robot Hierarchical Control System* by Anthony Barbera presents a detailed look at an NBS-developed architecture for applying computer controls to complex robot systems.

Hierarchical systems break the control of complex machine functions into a series of control levels, currently as many as three. Each control level accepts commands from the next higher level and interprets them as a series of simpler commands for the next lower level. Position and sensor information from the lower levels of the system are fed back up the "chain of command" through the same network.

A typical hierarchical control system for a robot arm might have simple servo controls at the lowest level, a higher control system capable of interpreting commands such as "grasp", "release", or "move", and a third level capable of guiding the arm through complex movements using sensory feedback data.

Hierarchical control offers several advantages, notably decreased programming time because comparatively few commands at the highest level are needed to produce a large number of simple steps at the lowest level, and a more efficient system design, since one high level unit may be used to control several lower levels in the hierarchy.

*An Architecture for a Robot Hierarchical Control System* provides a complete description of such a control system as developed at the National Bureau of Standards. In addition to describing the system and its possible uses, the report provides a documented listing of all the control programs for a three-level hierarchy control for a robot arm.

Published as NBS Special Publication 500-23, the book is available for $4.25 from the Superintendent of Documents, US Government Printing Office, Washington, DC 20402, USA. Ask for SD stock number 003-003-01874-1.

### Continuous flow analysis

The July/August issue of Technicon's *The AutoAnalyst* contains a report entitled *Aspects of Continuous Flow*, by Ralph Katzenell. He says that in the past decade a wealth of experience about the practical details of continuous flow has been amassed by regular Technicon users. This information has been used as the basis for a solid theoretical foundation. The author then proceeds to consider the theoretical aspects. For information about *The AutoAnalyst* contact The Editor, F. V. Hooley, Technicon Instruments Co. Ltd., Evans House, Hamilton Close, Basingstoke, Hants., England.

### Fluorescence spectrophotometry

Three new microprocessor systems for sorting data from Perkin-Elmer's high performance fluorescence spectrophotometers, Models MPF-4, 43A and 44A are described in detail in a new 12-page brochure. The Corrected Spectra Unit (Model CSU) corrects fluorescence exitation and fluorescence emission spectra which are then plotted on the standard recorder of the spectrophotometer.

The Differential Corrected Spectra Unit (Model DCSU-1) plots corrected fluorescence spectra and the difference between two spectra (either corrected or uncorrected spectra). Small differences can be expanded by factors of 2, 5, 10, 50, 100 and in addition, corrected synchronous excitation/emission spectra can be recorded. The Differential Corrected Spectra Unit (Model DCSU-2) performs the computation provided by the Model DCSU-1, plus first and second derivative curves of either corrected or uncorrected spectra. An electronic wavelength offset computation is applied to stored spectra points.

The Model DCSU-2 also performs the operations necessary for automatically recording fluorescence polarization, excitation spectra; anistropy calculations can also be made. The unit determines and stores the G factor (emission grating correction factor), when polarizers are placed before and after the sample in the spectrophotometer.

Fifteen examples of corrected spectra obtained with the three units are shown together with specifications on each unit. A free copy of the brochure is available from Perkin-Elmer Limited, Post Office Lane, Beaconsfield, Buckinghamshire, England.

### New specification for laboratory pH meters

BS 3145 *Laboratory pH meters*, a specification recently issued by the British Standards Institution, has a substantially different scope from the earlier edition of the standard (same number) which covered the now obsolete manually operated null or potentiometric instruments. This standard now deals with all types of laboratory pH meters in current production.

The new standard deals with definitions, presentation of the measured quantity, scale, errors of indication, input current, stability, glass electrode input, temperature compensating devices, set buffer adjustment and slope factor adjustment, and summarises the information to be given in literature provided.

Test methods included are for errors of indication, for determination of overall instrument error, for input current and for isopotential and temperature compensation. These compliance tests have been designed with typical usage of glass and reference electrodes in mind but simulated conditions are used to make the tests independent of electrode performance.

Copies of BS 3145, price £3.20, may be obtained from the BSI Sales Department, 101 Pentonville Road, London, N1 9ND, UK.

### Bibliographical references to publications

The British Standards Institution has published BS 5605 *Recommendations for citing publications by bibliographical references*. This standard, which recognizes both the systems of citation permitted by BS 1629 *Recommendations for bibliographical references*, the numeric system and the Harvard system, gives concise guidance to authors and editors on the preferred methods of arranging lists of references in books and journal articles; it includes methods of making attribution within the text. Copies of BS 5605, price £1.40, may be obtained from BSI Sales Department, 101 Pentonville Road, London N1 9ND, UK.

